# Natalizumab promotes anti-inflammatory and repair effects in multiple sclerosis

**DOI:** 10.1371/journal.pone.0300914

**Published:** 2024-03-25

**Authors:** Ragnhild Reehorst Lereim, Petra Nytrova, Astrid Guldbrandsen, Eva Kubala Havrdova, Kjell-Morten Myhr, Harald Barsnes, Frode S. Berven

**Affiliations:** 1 Proteomics Unit (PROBE), Department of Biomedicine, University of Bergen, Bergen, Norway; 2 Computational Biology Unit (CBU), Department of Informatics, University of Bergen, Bergen, Norway; 3 Department of Neurology and Center for Clinical Neuroscience, First Faculty of Medicine, Charles University and General University Hospital in Prague, Prague, Czech Republic; 4 Neuro-SysMed, Department of Neurology, Haukeland University Hospital, Bergen, Norway; 5 Department of Clinical Medicine, University of Bergen, Bergen, Norway; Fondazione Don Carlo Gnocchi, ITALY

## Abstract

**Background:**

Multiple sclerosis is an inflammatory and degenerative disease of the central nervous system leading to demyelination and axonal loss. Relapsing-remitting multiple sclerosis (RRMS) is commonly treated by anti-inflammatory drugs, where one of the most effective drugs to date is the monoclonal antibody natalizumab.

**Methods:**

The cerebrospinal fluid (CSF) proteome was analyzed in 56 patients with RRMS before and after natalizumab treatment, using label-free mass spectrometry and a subset of the changed proteins were verified by parallel reaction monitoring in a new cohort of 20 patients, confirming the majority of observed changes.

**Results:**

A total of 287 differentially abundant proteins were detected including (i) the decrease of proteins with roles in immunity, such as immunoglobulin heavy constant mu, chitinase-3-like protein 1 and chitotriosidase, (ii) an increase of proteins involved in metabolism, such as lactate dehydrogenase A and B and malate-dehydrogenase cytoplasmic, and (iii) an increase of proteins associated with the central nervous system, including lactadherin and amyloid precursor protein. Comparison with the CSF-PR database provided evidence that natalizumab counters protein changes commonly observed in RRMS. Furthermore, vitamin-D binding protein and apolipoprotein 1 and 2 were unchanged during treatment with natalizumab, implying that these may be involved in disease activity unaffected by natalizumab.

**Conclusions:**

Our study revealed that some of the previously suggested biomarkers for MS were affected by the natalizumab treatment while others were not. Proteins not previously suggested as biomarkers were also found affected by the treatment. In sum, the results provide new information on how the natalizumab treatment impacts the CSF proteome of MS patients, and points towards processes affected by the treatment. These findings ought to be explored further to disclose potential novel disease mechanisms and predict treatment responses.

## Background

Multiple sclerosis (MS) is a chronic disease of the central nervous system (CNS) causing widespread inflammation and neurodegeneration in the brain and spinal cord. The disease course is heterogeneous, and the rate and disability progression depend on both the subtype of the disease and on the therapy provided. Relapsing-remitting multiple sclerosis (RRMS) is the most common course, affecting about 85–90% of patients [1, 2]. Patients diagnosed with RRMS experience repeated episodes of CNS dysfunction, usually initially followed by partial or full remission. If not effectively treated, accumulating disability will usually appear along the disease course, and a substantial proportion convert to a secondary progressive course (SPMS) with gradual worsening without remission. Fewer patients (10–15%) experience a gradual worsening (without recovery) from the beginning of the disease, called primary progressive MS (PPMS) [3]. These clinical events are likely caused by disturbance in self-tolerance in peripheral immune cells that are recruited across the blood-brain barrier and damage CNS myelin, leading to disrupted neuronal signal conduction and clinical symptoms depending on the site of damage in the brain or spinal cord [2].

Several disease modifying therapies (DMTs) in RRMS have anti-inflammatory effects, thereby suppressing clinical relapses and disease progression. One such drug, natalizumab (Tysabri™), is a humanized monoclonal antibody that binds to the α4β1 integrin on leucocytes and hinders their recruitment across the blood brain barrier. Treatment with natalizumab effectively reduces inflammation and clinical relapses in the CNS [4] but is also associated with increased risk of opportunistic infections in the CNS, especially progressive multifocal leukoencephalopathy (PML) caused by the John Cunningham virus (JCV) [5]. Due to this risk, natalizumab is usually restricted to the treatment of more severe MS, or to JCV-negative patients [1, 6].

In this study, we used label-free LC-MS to analyze changes in the CSF proteome during natalizumab therapy after approximately two years of treatment. This provided insight into the effects of the drug on the CSF proteome and thereby also suggesting possible biomarkers for treatment response and mechanism of action in RRMS.

## Methods

### Patient selection

Approval for the study was given by the Ethics Committee of General University Hospital in Prague (Nr. 1976/17 S-IV) and written informed consent was obtained from all patients. For the Bergen location, the study was approved by the Regional Committee for Research Ethics (REK Sør/Øst, REK Vest) with approval number 2018/120.

**Fig 1:**
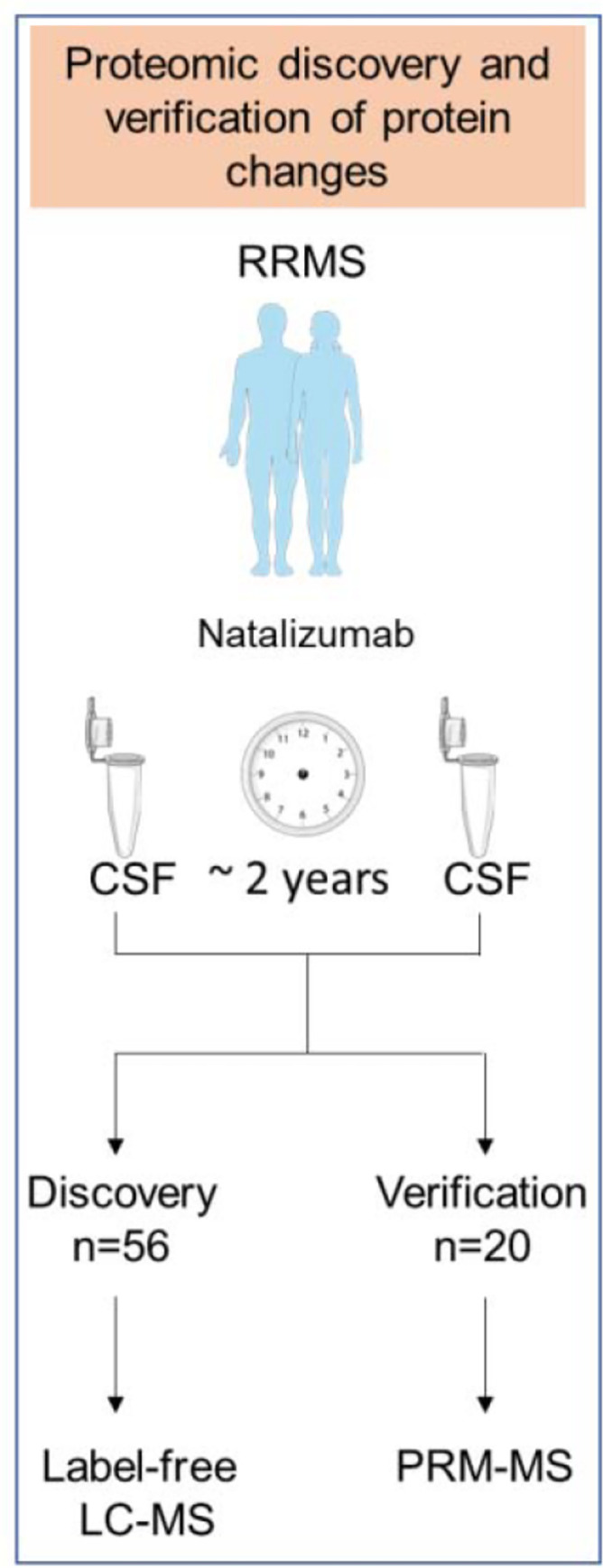
Overview of participants, study design, and experiments. Paired CSF patient samples from 76 individuals were collected prior to or at initiation of natalizumab infusions, and after about two years of therapy. A total of 56 patients were included in a label-free LC-MS discovery study and 20 in a PRM-MS verification study.

The study included paired CSF patient samples from 76 individuals collected prior to or at initiation of natalizumab infusions, and after about two years of therapy. The second sample was taken either during treatment with natalizumab, or during the natalizumab wash-out period of maximum 2.5 months after the last natalizumab infusion. A total of 56 patients were included in the discovery study and 20 in the verification study (Fig 1). Lumbar puncture (LP) was performed due to safety reasons because the assessment of JCV index was not available at that time. LPs in all studied patients were performed via an atraumatic needle at the MS center, at the Department of Neurology, General University Hospital in Prague. All samples were stored at -80˚C.

All patients started natalizumab treatment for disease activity (clinical and/or on brain/spinal cord MRI). The majority of patients had received DMT when first sampled. The largest group of patients (n = 39) had received interferon beta for the period prior to the first sample collection (Table 1). The patients treated with interferon beta were split across the discovery and verification studies (19 in the discovery study, 20 in the verification study). Approval for the study was given by the institutional ethical committees and written informed consent was obtained from all patients. The samples were accessible from March 13^th^ 2018 for this study. None of the authors individually possessed all the identification keys needed to link the proteomics data to the patient identity during the study.

**Table 1:** Overview of patients in the discovery and verification cohorts.

*Cohort*	*Drug at baseline*	*n*	*Sex* *F/M*	*Age* *Median (±SD)*	*Years between sampling Median* *(min-max)*	*Total relapses Median (min-max)*	*Relapses between sampling Median (min-max)*	*Protein concentration baseline (average ug/ul ± SD)*	*Protein concentration follow-up (average ug/ul* *± SD)*
Discovery	Interferon-beta (19), no therapy (10), natalizumab (9) ^#^, azathiopine and interferon-beta (8), glatiramer acetate (6), immunoglobulin (4)	56	42/14	33.5±10	2.3 (0.8–4.6)	7 (1–27)	1 (0–4)	0.38±0.17	0.6±0.20
Verification	Interferon-beta (20)	20	14/6	38±11	2.3 (1.2–3.8)	7 (1–22)	1 (0–5)	0.75±0.10	0.71±0.10

Age is at initiation of natalizumab treatment.

^#^ Includes patients taking a break from the natalizumab treatment due to pregnancy, which notably also largely explains the wide time range between samplings.

Note that one patient was excluded from the verification experiment due to consistently irregular results as determined by hierarchical clustering of fold changes.

### Sample preparation

The protein concentration for each sample was measured in duplicate by BCA (Thermo). Aliquotes of 5 μg (discovery) and 10 μg (verification) were lyophilized dry and trypsinated by in-solution digestion and desalted as previously described [7]. The peptides were solved for LC- MS/MS in 2% ACN, 1% FA. Five μg (discovery) and 10 μg (verification) sample pools served as quality controls and were prepared by combining equal volume of samples in each experiment.

The patients in the discovery study were randomized according to sex, treatment at the baseline, and the time between sampling throughout the sample preparation and LC-MS analysis. To avoid introducing technical variation between samples from the same patient, paired samples were processed directly after each other, alternating the baseline and follow-up sample throughout the protocol. Quality controls were combined and run each 12–14 sample to help with sample alignment and to control technical variability during LC-MS/MS.

### Liquid chromatography

#### Discovery study

Peptides were injected into an Ultimate 3000 RSLC system (Thermo Scientific, Sunnyvale, California, USA) connected to a Q Exactive HF equipped with a nanospray Flex ion source (Thermo Scientific, Bremen, Germany). The samples were loaded on an Acclaim PepMap 100, 2cm x 75μm i.d. nanoViper pre-column, packed with 3μm C18 beads at a flow rate of 5μl/min for 5 min with 0.1% TFA (trifluoroacetic acid, vol/vol). Peptides were separated during a biphasic ACN gradient from two nanoflow UPLC pumps (flow rate of 0.250 μl/min) on a 50 cm analytical column (Easy-Spray 803, 50cm x 75μm i.d. PepMap RSLC column, packed with 2μm C18 beads (Thermo Scientific). Solvent A was 0.1% FA (vol/vol) in water, and solvent B was 100% ACN, 0.1% FA. The following gradient was used; 0 min 5% B, 5 min 5% B, 140 min 35% B, 155 min 80% B, 170 min 80% B, 175 min 5% B, 195 min 5% B. Samples were a maximum of 48 hours in the autosampler at 10˚C prior to injection.

#### Verification study

Peptides were separated on an analytical column PepMapTM RSLC C18 (diameter width 75 μm x 25 cm, particle size at 2 μm and 100 A in length) with the combination of 95% solvent A (0.1% FA) and 5% solvent B (100% ACN, 0.1% FA) with a flow rate of 0.250 μl/min. The column gradient for peptide elution went from 0–5 min with 5% solvent B, then an increase at 5–5.5 min to 7% of solvent B, 5.5–65 min 22% B, 65–87 min 35% B, 87–92 min 90% B and 92–102 min with 90% B. At 102–105 min solvent B decreased to 5% solvent B and held a 5% solvent B from 105–120 min.

### Mass spectrometry

#### Discovery study

The Q Exactive HF mass spectrometer was controlled through Tune 2.9 and Xcalibur 3.0. The method duration was 195 min (runtime 10–185 min), and the mass spectrometer was operated in data dependent acquisition (DDA) mode and switched between full scan MS^1^ and MS^2^ acquisition. MS^1^ spectra were acquired in profile mode in the scan range of 375−1500 m/z with resolution of R = 120 000, automatic gain control (AGC) target of 3e^6^, and a maximum injection time (IT) of 100 milliseconds. Dynamic exclusion was set to 25 seconds, and isotope exclusion was turned on. Lock-mass (445.12003 m/z) internal calibration was used. The top 12 intensity precursors over an intensity threshold of 5e^4^ were sequentially isolated for MS^2^ with a window of 1.6 m/z with 0.3 m/z isolation offset. Precursors were sampled to a target AGC value of 1e^5^, or to an IT of 110 milliseconds. The precursors were fragmented by higher-energy collision dissociation (HCD) at a normalized collision energy of 28%. The MS^2^ resolution was R = 30 000. The MS^2^ spectra were acquired in centroid mode.

#### Verification study

The instrument was controlled through Q Exactive HF Tune 2.4 and Xcalibur 3.0. The mass spectrometer was operated in PRM scheduled mode and switched between full scan MS^1^ between every 12th PRM MS^2^ scan. MS^1^ spectra were acquired in profile mode in the scan range of 375−1500 m/z with resolution of 15 000, automatic gain control (AGC) target of 3e^6^, and a maximum injection time (IT) of 15 milliseconds. The target peptides on the inclusion list were sequentially isolated for higher-energy collision dissociation (HCD) fragmentation and MS^2^ acquisition with optimized collision energies. The method duration was 120 min (runtime 10–110 min). MS^2^ spectra were acquired with resolution R = 60 000 at 200 m/z, IT of 118 ms, AGC target value at 2e^5^ and precursor isolation window was set to 0.7 m/z.

### Statistical analysis

The label-free analysis was run on three analytical columns and searched collectively in MaxQuant v1.5.2.8 [8, 9]. The search parameters were as default with the following exceptions: enzyme set to trypsin; LFQ quantification enabled, and LFQ min ratio count set to 1; variable modifications included oxidation of methionine and acetylation of protein N-terminal, and carbamidomethylation of cysteine as a fixed modification. The protein sequence database was the reviewed proteome from UniProtKB/Swiss-Prot downloaded 31/3-2019, and the search included contaminants from the MaxQuant contaminants database. In total, 1431 proteins were quantified using label-free DDA LC-MS/MS, and after filtering out contaminants, decoy hits, and proteins labelled “only identified by site”, 1304 proteins remained. From these, only the 1164 proteins found in two or more pairs of patient samples were analyzed further.

The individual LFQ normalized protein intensity values from MaxQuant were log_2_-transformed, and the change in abundance from before to after treatment calculated by subtracting the protein abundance after treatment by the protein abundance before treatment, yielding a total of 56 fold changes per protein, *i*.*e*., one per patient. The median fold change for each protein was used as a representative abundance change for the respective protein, and the percentage of the fold changes that were either positive or negative was calculated.

A paired *t*-test of the normalized intensity values of the 1164 quantified proteins, followed by Benjamini-Hochberg correction for multiple testing with a significance threshold of 0.01, revealed 287 significantly differentially abundant proteins between the samples collected before and after treatment. The distribution of the median fold changes in the dataset was greatly affected by immunoglobulins (proteins matching to UniProt keyword “Immunoglobulin” (KW-1280)) and these 57 proteins were therefore excluded from the following analysis (S1 Fig). To identify the proteins that changed the most, a fold change cut-off was calculated using the *z*-score (<0.05) on the median log_2_ fold change. All significantly differentially abundant proteins (including the immunoglobulins) were included in the gene ontology enrichment analysis and in the comparison to CSF-PR [10].

### Comparison with public data in CSF-PR

The proteins in the discovery dataset were separated into the following categories: increased (FDR<0.01, log_2_ fold change >0), decreased (FDR>0.01, log_2_ change<0), and equal (FDR>0.01), after natalizumab treatment, and the three categories were compared to all datasets in the online database CSF-PR [10] that compared RRMS to other neurological diseases (OND), chosen due to the lack of available CSF data from patients without neurological diseases. The result of this comparison were then grouped into the following categories: (i) *opposite*–proteins increased during treatment with natalizumab, but were decreased in RRMS vs. controls from the studies in CSF-PR or vice versa; (ii) *equal*–proteins increased after treatment with natalizumab and also increased in RRMS vs. controls from the studies in CSF-PR, or decreased after treatment with natalizumab and also decreased in RRMS vs. controls from the studies in CSF-PR; (iii) *Natalizumab only–*proteins increased or decreased during treatment, but unaffected in RRMS vs. controls from the studies in CSF-PR; (iv) *RRMS only*–proteins increased or decreased in RRMS vs. controls from the studies in CSF-PR, and not affected by natalizumab treatment; and finally (v) *not changed*–proteins not changed in any comparison. The goal of this comparison was to investigate if the proteome changes in natalizumab countered protein abundance changes in the CSF frequently observed in RRMS, otherwise only possible by an extensive literature search.

### Network and gene ontology analysis

The proteins in each of the five CSF-PR comparison categories were searched in stringApp v1.6.0 [11] and annotated by BiNGO (v3.0.4) [12] through Cytoscape v3.8.2 [13]. The default STRING confidence score of 0.4 was set for minimum interaction confidence, and all interactions were allowed. For functional annotation by Gene Ontology Biological Process (GOBP), the full protein set of the 1164 quantified proteins were used as background for the enrichment. Gene ontology and human annotations were downloaded 25^th^ of February 2021. GOBP evidence codes IEA (Inferred from Electronic Annotation) were excluded. GOBP term overrepresentation was estimated by a hypergeometric test, followed by Benjamini-Hochberg false discovery rate correction, with GOBP terms with an FDR<0.05 considered as significant.

### Peptide selection for verification by parallel reaction monitoring

The proteins changing the most during treatment, as determined by statistical significance (FDR<0.01), fold change (*z*-score <0.05) and percentage of patients that were changed in the same direction as the median (>80%), were considered the most interesting and assessed for verification by parallel reaction monitoring (PRM). Additionally, immunoglobulins, proteins of special interest due to the disease (*e*.*g*., CHI3L1, CHI3L and CHIT) and proteins of special interest from the comparison with CSF-PR were also included.

Peptide selection was initially based on the peptide information in the discovery study. Briefly, peptide intensities were normalized by median subtraction after log_2_ transformation at the sample level, followed by a paired *t*-test using a *p*-value threshold of 0.05. The UniProt knowledgebase was used to determine the likelihood of modifications for each peptide, avoiding peptides with known post-translational modifications and peptides containing cysteine and methionine. Accordingly, peptides were selected based on peptide intensity, statistical significance, peptide uniqueness and possible modifications. A mixture of Thermo pepotec peptides, Thermo AQUA peptides and JPT peptides were used in the PRM verification experiment, with each protein being represented by one to three peptides. In total, 94 peptides representing 62 proteins were included in the verification experiment, and of these, 79 peptides from 54 proteins were used in the final assay (S3 Table).

### Fragment selection, peak integration, and general data analysis for PRM

The peptides were analyzed in Skyline version 20.0.31 [14, 15]. The fragments were initially selected to form a library created from the discovery data and refined to avoid fragments with clear interference as determined by visual inspection and/or high ppm (>10 ppm). Collision energy optimization was performed in duplicates for CE 17, 23, 28 and 30, thus influencing the fragment selection. Each peptide was quantified using a minimum of three fragments. The peak integration was determined by Skyline, with limited manual refinement. In instances with peak truncation, the results from the replicate with a non-truncated peak were considered representative and used in the downstream analysis. Measurements with less than eight points across the chromatographic peak were excluded.

### Calibration curves

Nine-point reverse calibration curves were prepared with variable spike-in levels of heavy peptide in the verification pools. These were prepared in trypsinated duplicates. Briefly, the calibration curves were prepared as a dilution series covering 2.4 orders of magnitude, spanning from eight times the estimated 1:1 concentration, down to 0.03 of the estimated 1:1. Following refinement in Skyline, the results were exported for analysis in R [16]. A weighted linear model was fitted to model the relationship between the ratio to standard and theoretical concentration (fmol/ul injected), taking into account the higher variability in the high concentration area, as is commonly done (weights = 1/theoretical concentration) [17]. The linear area was determined by manual inspection of the fitted line, including the low concentration area.

### Data analysis of verification data

Following integration in Skyline, the data was exported for analysis in Microsoft Excel and R. Briefly, measurements below the linear area as determined by the calibration curve were filtered out. Subsequently, the peptide concentration (fmol/ul sample) was log_2_ transformed, and the average for each trypsinated replicate calculated. If only one replicate had a valid value, it was considered representative and used in the downstream analysis. Paired *t*-tests were performed, and a Benjamini-Hochberg FDR correction of 0.05 applied. In R, the graphics package ggplot2 [18] was used for graphical presentation of the results.

## Results

**Fig 2:**
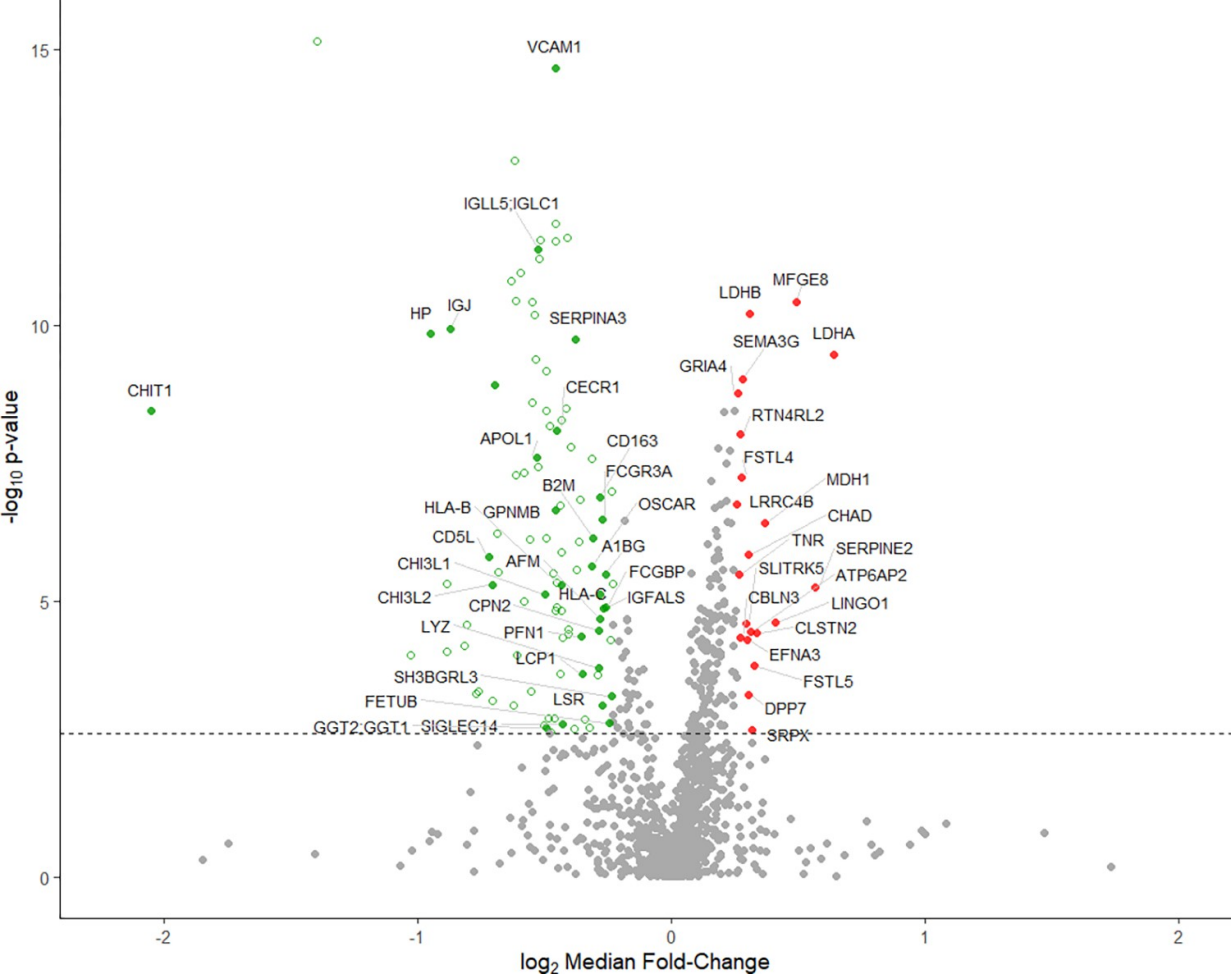
Proteins most affected by natalizumab treatment. Volcano plot of the log_10_ *p*-value and log_2_ median fold changes for all proteins in the dataset. The 57 proteins most affected by the treatment (FDR<0.01, *z*-score <0.05) are colored. Filled red (increased) and green (decreased) dots represent the significantly differentially abundant proteins with the highest median fold change defined with *z*-score significance < 0.05 (after excluding immunoglobulins). Proteins are labelled by their gene short name. Antibody-immunoglobulins (FDR<0.01) are shown as green unfilled dots (see Fig 3).

The discovery (n = 56 patients) analysis by label-free LC-MS quantified 1164 CSF proteins, of which 287 were significantly differentially abundant after treatment with natalizumab (paired two-sided *t*-test, FDR<0.01) (Fig 2). Overall, natalizumab treatment resulted in a decrease in inflammatory proteins and an increase in proteins with neurological function. Proteins related to angiogenesis, metabolism, and some additional processes (as further explored below) were also found changed. Furthermore, the analysis showed that the protein abundance changes during natalizumab treatment were consistent across patients for a large portion of the proteins. Eighty of the significantly changed proteins were for example reduced or increased in over 80% of the patients (Tables 2 and 3).

**Fig 3:**
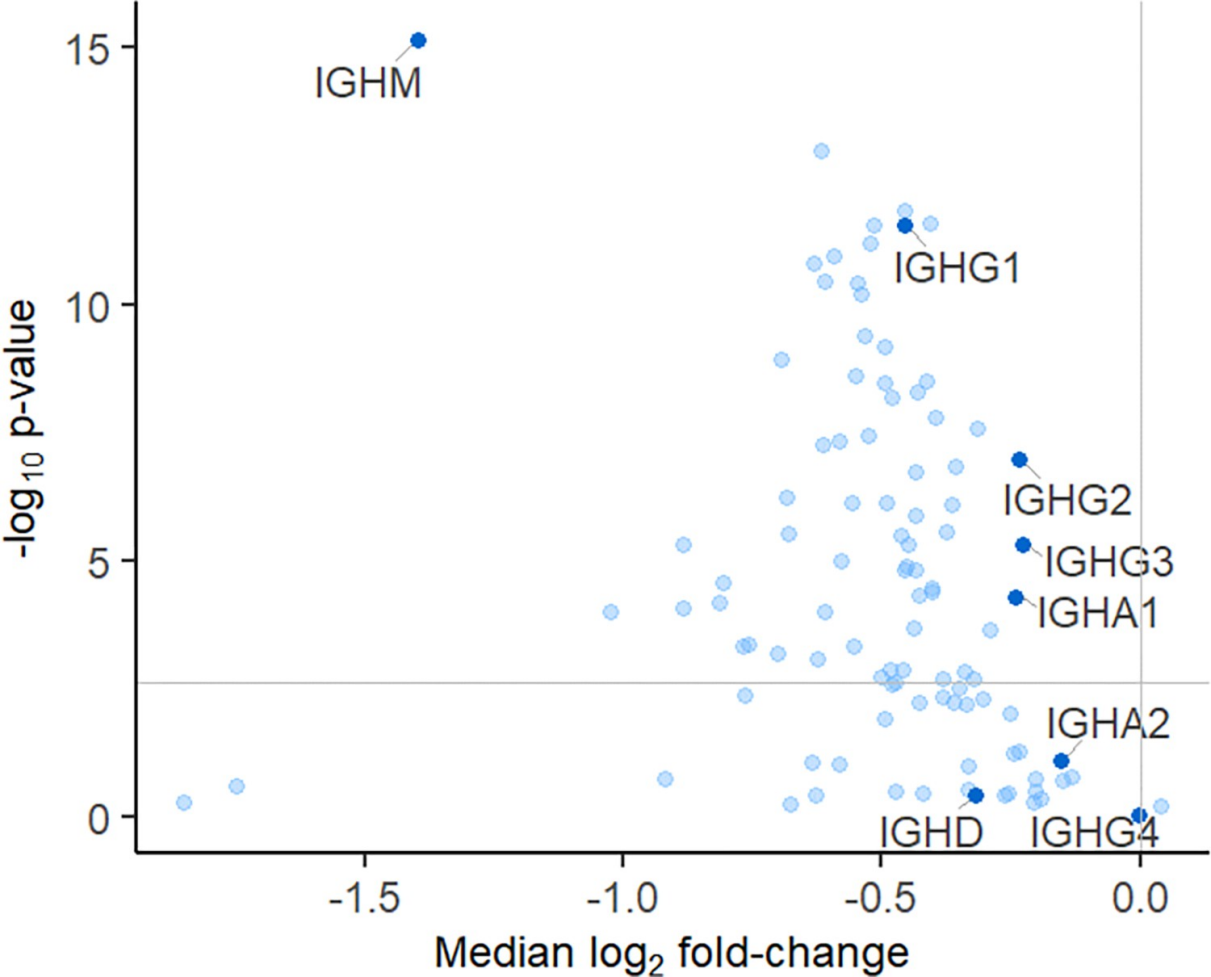
Change in the abundance of immunoglobulins targeted for somatic rearrangement (antibodies) during treatment with natalizumab. Proteins above the horizontal line are considered significantly differently abundant (FDR<0.01). Constant heavy chains (dark blue) are labelled by their gene short names.

**Table 2:** Immunoglobulins that were the most affected by the treatment (FDR<0.01) and changed in the majority of patients (>80%), excluding protein groups.

*Accession*	*Protein name*	*Gene name*	*Unique peptides*	*% blanks* **	*Median* *log*_*2*_ *FC*	*% changed* *
*P01871*	Ig mu chain C region	IGHM	33	0	-1.40	95
*P01624*	Ig kappa chain V-III region POM	IGKV3-15	0	0	-0.59	95
*P06310*	Ig kappa chain V-II region RPMI 6410	IGKV2-30	3	0	-0.62	93
*P01780*	Ig heavy chain V-III region JON	IGHV3-7	7	0	-0.51	91
*P01834*	Ig kappa chain C region	IGKC	17	0	-0.46	89
*P01857*	Ig gamma-1 chain C region	IGHG1	18	0	-0.46	89
*P06312*	Ig kappa chain V-IV region	IGKV4-1	7	0	-0.55	89
*A0A0C4DH38*	Immunoglobulin heavy variable 5–51	IGHV5-51	3	0	-0.43	89
*A0A0C4DH68*	Immunoglobulin kappa variable 2–24	IGKV2-24	3	11	-0.56	88
*P01599*	Ig kappa chain V-I region Gal	IGKV1-17	2	0	-0.63	88
*P01602*	Ig kappa chain V-I region HK102	IGKV1-5	3	0	-0.61	88
*A0A075B6K4*	Immunoglobulin lambda variable 3–10	IGLV3-10	5	0	-0.49	88
*A0A0C4DH72*	Immunoglobulin kappa variable 1–6	IGKV1-6	0	0	-0.61	88
*P01704*	Ig lambda chain V-II region TOG	IGLV2-14	1	9	-0.49	86
*A0A075B6S2*	Immunoglobulin kappa variable 2D-29	IGKV2D-29	2	0	-0.52	86
*P0DOY3*	Immunoglobulin lambda constant 3	IGLC3	2	0	-0.41	86
*P06331*	Ig heavy chain V-II region ARH-77	IGHV4-34	1	0	-0.48	86
*P01705*	Ig lambda chain V-II region NEI	IGLV2-23	2	50	-0.82	86
*A0A075B6S5*	Immunoglobulin kappa variable 1–27	IGKV1-27	3	18	-0.81	85
*A0A075B6R2*	Immunoglobulin heavy variable 4–4	IGHV4-4	2	7	-0.58	85
*A0A0B4J1Y9*	Immunoglobulin heavy variable 3–72	IGHV3-72	6	0	-0.41	84
*P01619*	Ig kappa chain V-III region B6	IGKV3-20	2	0	-0.53	84
*A0A0B4J1U7*	Immunoglobulin heavy variable 6–1	IGHV6-1	5	0	-0.39	84
*A0A087WSY4*	Immunoglobulin heavy variable 4-30-2	IGHV4-30-2	3	34	-0.88	84
*A0A0B4J1X8*	Immunoglobulin heavy variable 3–43	IGHV3-43	2	34	-0.38	84
*P80748*	Ig lambda chain V-III region LOI	IGLV3-21	1	48	-0.44	84
*A0A0C4DH24*	Immunoglobulin kappa variable 6–21	IGKV6-21	1	0	-1.02	83
*A0A0C4DH25*	Immunoglobulin kappa variable 3D-20	IGKV3D-20	2	12.5	-0.44	82
*A0A0G2JS06*	Immunoglobulin lambda variable 5–39	IGLV5-39	1	7	-0.43	81
*P01706*	Ig lambda chain V-II region BOH	IGLV2-11	3	0	-0.31	80
*P01859*	Ig gamma-2 chain C region	IGHG2	15	0	-0.24	80
*A0A075B6K5*	Immunoglobulin lambda variable 3–9	IGLV3-9	2	0	-0.36	80
*A0A0B4J1V0*	Immunoglobulin heavy variable 3–15	IGHV3-15	6	0	-0.37	80
*P01742*	Ig heavy chain V-I region EU	IGHV1-69	2	0	-0.43	80
*A0A0B4J1V2*	Immunoglobulin heavy variable 2–26	IGHV2-26	7	0	-0.88	80
*A0A0C4DH29*	Immunoglobulin heavy variable 1–3	IGHV1-3	3	55	-0.71	80
*P01764*	Ig heavy chain V-III region VH26	IGHV3-23	2	46	-0.50	80

Ordered by the percentage of patients where the protein was changed.

* % of patients with protein increased/decreased (log_2_ FC >0/<0) in direction of median.

** % of patients where the immunoglobulin were not detected in at least one sample.

**Table 3:** Proteins that were the most affected by the treatment (FDR<0.01, *z*-score<0.05) and changed in the majority of patients (>80%), excluding antibody immunoglobulins and protein groups.

*Accession*	*Protein name*	*Gene name*	*Unique peptides*	*% blanks*	*Median log*_*2*_ *FC*	*% changed* *
*P01591*	Immunoglobulin J chain	IGJ	11	0	-0.87	95
*P19320*	Vascular cell adhesion protein 1	VCAM1	22	0	-0.45	93
*P00738*	Haptoglobin	HP	22	0	-0.95	93
*Q08431*	Lactadherin	MFGE8	12	2	0.49	91
*P01011*	Alpha-1-antichymotrypsin	SERPINA3	41	0	-0.38	89
*P07195*	L-lactate dehydrogenase B chain	LDHB	19	0	0.31	86
*Q9NS98*	Semaphorin-3G	SEMA3G	21	0	0.28	86
*P48058*	Glutamate receptor 4	GRIA4	16	0	0.26	86
*Q86VB7*	Scavenger receptor cysteine-rich type 1 protein M130	CD163	35	0	-0.28	86
*Q9NZK5*	Adenosine deaminase CECR1	CECR1	11	0	-0.45	86
*Q13231*	Chitotriosidase-1	CHIT1	25	13	-2.05	86
*Q14956*	Transmembrane glycoprotein NMB	GPNMB	4	4	-0.45	85
*P00338*	L-lactate dehydrogenase A chain	LDHA	13	0	0.64	84
*Q9NT99*	Leucine-rich repeat-containing protein 4B	LRRC4B	23	0	0.26	84
*P07093*	Glia-derived nexin	SERPINE2	10	9	0.57	82
*P40925*	Malate dehydrogenase, cytoplasmic	MDH1	12	0	0.37	82
*Q9UHL4*	Dipeptidyl peptidase 2	DPP7	13	0	0.31	82
*Q6MZW2*	Follistatin-related protein 4	FSTL4	30	0	0.28	82
*Q86UN3*	Reticulon-4 receptor-like 2	RTN4RL2	11	0	0.27	82
*Q9Y6R7*	IgGFc-binding protein	FCGBP	101	0	-0.27	82
*O14791*	Apolipoprotein L1	APOL1	18	0	-0.53	82
*Q9H4D0*	Calsyntenin-2	CLSTN2	7	5	0.34	81
*O94991*	SLIT and NTRK-like protein 5	SLITRK5	2	7	0.30	81
*P35858*	Insulin-like growth factor-binding protein complex acid labile subunit	IGFALS	27	0	-0.26	80
*Q15782*	Chitinase-3-like protein 2	CHI3L2	12	11	-0.70	80

Ordered by the percentage of patients where the protein was changed.

* % of patients with protein increased/decreased (log_2_ FC >0/<0) in direction of median.

### Proteins decreased in abundance after treatment

A high number of the quantified proteins were immunoglobulins (101 of 1164), and the majority of these significantly decreased after treatment (Fig 3). Immunoglobulin heavy constant mu (IGHM) was decreased, confirming previous proteomic studies [17]. In fact, IGHM decreased during treatment in 95% of the paired patient samples and had the greatest fold change of the detected immunoglobulins. Furthermore, we found that the heavy chains of IgG subclasses 1, 2 and 3 (IGHG1, IGHG2, IGHG3) and IgA1 (IGHA1) decreased during treatment, indicating a reduction in humoral adaptive immunity following natalizumab treatment (Fig 3).

As the immunoglobulins highly affected the fold-change distribution in the dataset (S1 Fig), a fold change cutoff was calculated on the dataset excluding the immunoglobulins, yielding 57 non-immunoglobulin proteins that were considered highly affected by natalizumab treatment (Fig 2). CHIT1, chitinase3-like protein 1 (CHI3L1) and VCAM1 were among the proteins that decreased, confirming previous studies [17, 19].

The following immune active proteins were also found decreased after treatment with a significant fold-change: β2-microglobulin (B2M), HLA-B and HLA-C (all antigens in the HLA-class 1 complex to CD8 T -cells), acute phase proteins haptoglobin (HP), and scavenger receptor cysteine-rich type 1 protein M130 (CD163). Furthermore, APOL1, LSR [20] and APOA 1 [21], all involved in lipid transport, also decreased during natalizumab treatment.

### Proteins increased in abundance after treatment

In the opposite direction, the protein with the lowest *p*-value, and the third most increased in median fold change, was lactadherin (MFGE8), known to have roles in both neuron development and angiogenesis [22, 23].

Lactate dehydrogenase A and B (LDHA, LDHB) had the first and tenth highest median fold change increase after treatment, respectively. Also malate dehydrogenase (MDH1), with the fifth highest median fold change increase, active in the malate-aspartate shuttle necessary for regeneration of NADH in the mitochondria [24], was found increased after treatment.

Among the other proteins with the highest fold change increase were renin receptor (ATP6AP2), with roles in both blood pressure regulation [25] and synaptic transmission in the CNS [26], glutamate receptor 4 (GRIA4) and leucine-rich repeat-containing protein 4B (LRRC4B), the latter two indicating an increase in excitatory signaling in the brain following treatment. Other proteins found with positive fold changes that regulate (promote or suppress) neurite outgrowth, were SERPINE2 (promotes), SLITRK5 (suppresses), RTN4RL2 (suppresses).

Furthermore, leucine-rich repeat and immunoglobulin-like domain-containing nogo receptor- interacting protein 1 (LINGO1), an inhibitor of oligodendrocytal differentiation and axonal myelination [27], was also found among those most increased in median fold change. Inhibiting this protein from interacting with its receptors has resulted in improved recovery in the MS animal model EAE [28, 29]. A monoclonal antibody inhibiting LINGO-1 (opicinumab) however showed no clear improvement in phase II trials in MS [30], or in patients with their first acute optic neuritis incident though it showed improvement in visual evoked potentials [31].

### Relation between RRMS pathogenesis and proteins affected by the treatment

**Fig 4:**
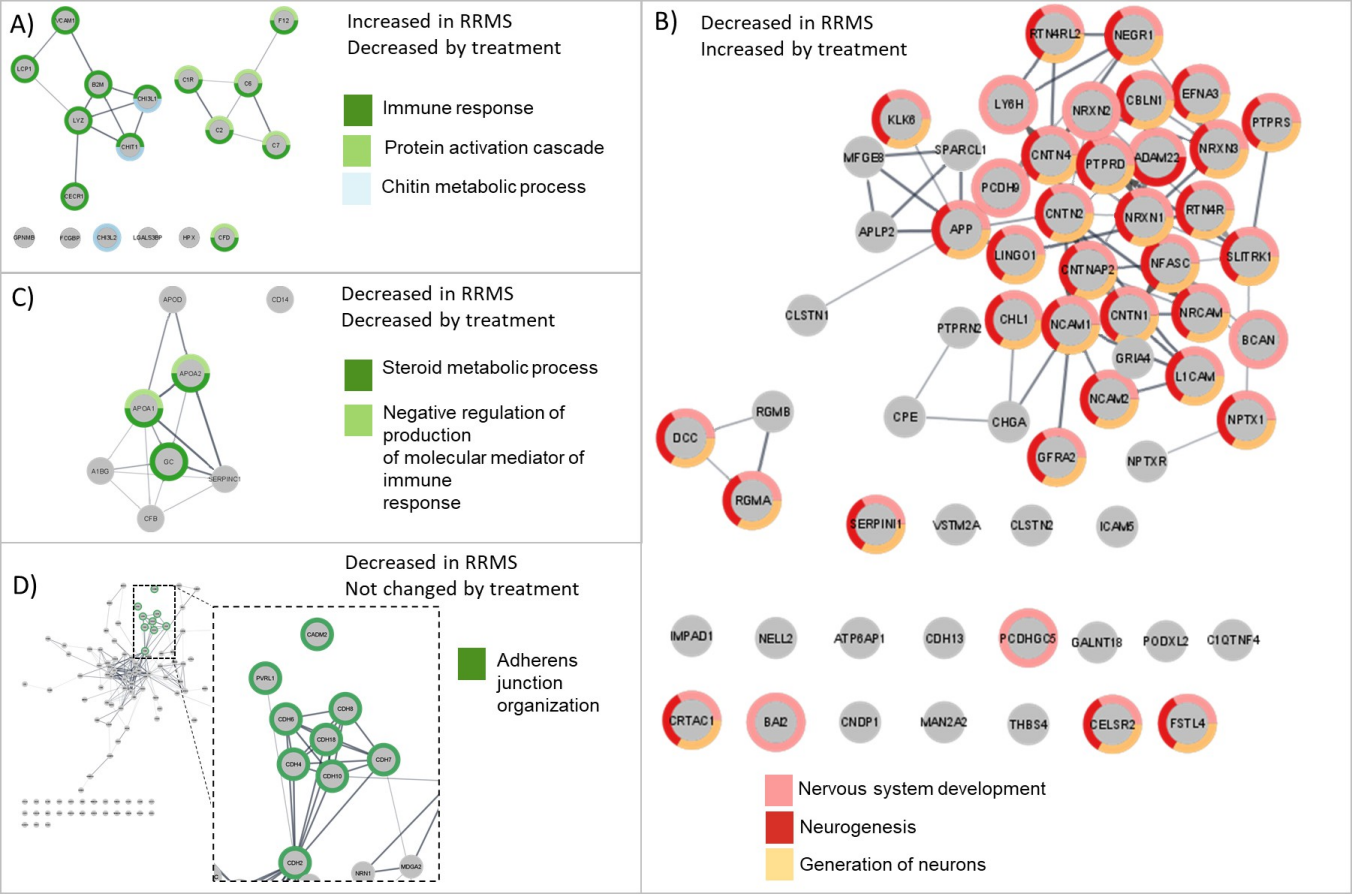
Determination of disease relevance of significantly differentially abundant proteins by comparison to RRMS vs. OND datasets in CSF-PR. Proteins in GOBP-enriched terms (FDR<0.05) are color-coded as indicated. The edge widths are proportional to the STRING scores. (A) Proteins found decreased by natalizumab that are commonly increased in RRMS patients compared to neurological controls have immune and chitin metabolic activity. (B) Proteins found increased by natalizumab that are commonly decreased in RRMS compared to neurological controls have neurological activity. (C) Proteins that are known to decrease in RRMS compared to neurological controls and decreased during treatment have steroid metabolic process activity. D) Proteins not changed by treatment that decrease in RRMS have adherence junction organization activity. Picture: Cytoscape, stringApp.

To determine if the 287 proteins found changed after treatment with natalizumab were involved in processes previously found by proteomics to be affected by RRMS, the 287 proteins were compared to proteins changed in public datasets in CSF-PR comparing RRMS vs. OND [10]. This approach would also reveal the effects of natalizumab on RRMS biomarker candidates from previous studies (S1 Table). The biomarker categories from CSF-PR and our data that were deemed most relevant to compare are outlined in Table 4. The two most significant GOBP terms representing overlapping proteins for each category are also listed in this table. Notably, a network analysis was performed on the proteins in the categories with a significant GOBP and is summarized in Fig 4.

**Table 4:** Comparison of proteins affected/not affected by the treatment to public data comparing RRMS to neurological controls in CSF-PR.

Interpretation		RRMS	Treatment	#Proteins	Top 2 affected GOBP
	Efficient	↑	↓	22	Immune response Immune effector process
**Opposite**	immunotherapy and repair	↓	↑	61	Nervous system development Generation of neurons
		↑	↑	11	No significant GOBP terms
**Equal**	Possible ongoing disease activity	↓	↓	8	Steroid metabolic process Neg. reg. of production of molecular mediator of immune response

In total, 22 proteins reported increased in RRMS compared to controls in CSF-PR were decreased after treatment with natalizumab from our data (Fig 4A). The resulting STRING interaction network of these 22 proteins contained two clusters, one including CHIT1, CHI3L1 and other known inflammatory proteins, the other including complement factors. This observation corresponds with the well-known anti-inflammatory effects from natalizumab therapy. Note that there were four immunoglobulins in this category for which the accession numbers could not be matched by STRING which are therefore not part of the network. In addition, 18 immunoglobulins with highly decreased abundance from the treatment study did not match any accession number in CSF-PR.

On the other hand, 61 proteins that have been reported decreased in RRMS compared to controls in CSF-PR were increased after treatment in our data (Fig 4B), with the key biological processes affected being *nervous system development* and *generation of neurons*. The resulting STRING network indicates several separated clusters of proteins mainly linked to GOBP processes concerning neurons, axons, and synapses, possibly involved in neurodegeneration. As these proteins were decreased in RRMS and increased after treatment, they appear to be positively affected by the natalizumab treatment.

Other interesting proteins include those that previously have been suggested to be affected by RRMS, and that decreased/increased similarly during treatment in our data, since they might be involved in disease processes that are not affected by the therapy. Eight proteins have been reported decreased in RRMS compared to controls and were also decreased during treatment in our data (Fig 4C). All of these, except for CD14, are connected in the STRING analysis and with *steroid metabolic process* as the most affected biological process. This category includes vitamin-D binding protein (GC), apolipoprotein A-I (APOA1) and apolipoprotein A-II (APOA2) that were annotated with the majority of enriched GOBP annotations. In particular, APOA1 and APOA2 were part of an enriched gene set annotated with terms linked to negative regulation of production of molecular mediator of immune response, regulation of very-low density lipoprotein particle remodeling and protein oxidation, among others.

Similarly, 11 proteins previously reported increased in RRMS compared to controls were still increased after treatment in our data. However, there were no well-connected interaction networks for this category, and no GOBP enriched terms. As these proteins are further increased after treatment, they can potentially be part of on-going disease activity not affected by the natalizumab treatment.

The proteins not affected by the treatment were also included in the CSF-PR analysis. Of these, 108 were decreased in RRMS compared to controls, with the top biological process from the GOPB analyses being “adherence junction organization”, represented by a closely connected cluster of cadherins (Fig 4D).

### Verification of proteins changed by natalizumab treatment

**Fig 5:**
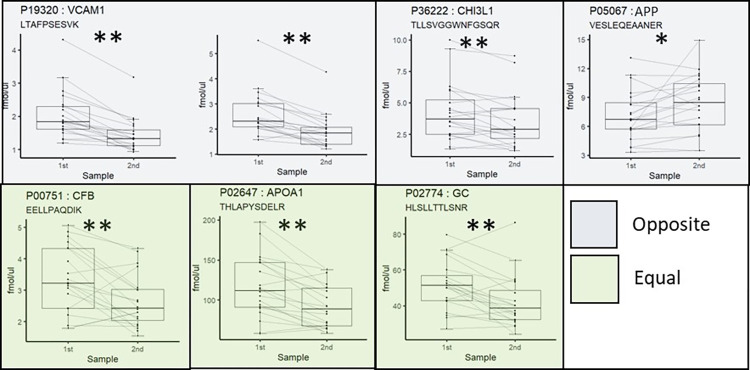
Examples of the verified protein changes in natalizumab treatment and their relevance to RRMS. Boxplots of the peptide concentration of the first and the follow-up sample, with whiskers indicating the largest and smallest value greater than or less than 1.5 times the inter quartile range from the hinges. Lines connect samples from one patient. The change in protein abundance as compared to published data in CSF-PR comparing RRMS to controls is indicated by the color coding. * = FDR<0.05, ** = FDR<0.01. An overview of all proteins measured in each CSF-PR category can be found in Table 6.

Seventy-nine peptides representing 54 proteins were included in the PRM verification process. Similar to the discovery study, the results from the verification study showed fold changes that were moderate, with low *p*-values. An overview of all the tested peptides and proteins can be found in S3 Table, and the results of the verification experiments for the 21 most changed proteins are listed in Table 5. Table 6 shows an overview of the verification results for proteins in the most relevant CSF-PR categories, and all categories in S2 Table. Fig 5 shows boxplots of the measured peptide concentration of the first and the follow-up sample for a selection of the verified proteins.

**Table 5:** Verification of the most changed proteins from the discovery study measured in new patients by PRM-MS.

Accession	Protein	Gene	Peptides	Median log_2_ FC	95% CI	FDR	% Changed ^a^
*P19320*	Vascular cell adhesion protein 1	VCAM1	LTAFPSESVK SLEVTFTPVIEDIGK	-0.44 -0.40	[-0.35, -0.65] [-0.28, -0.58]	** **	100.0 95.0
*P01591*	Immunoglobulin J chain	IGJ	SSEDPNEDIVER IVLVDNK CYTAVVPLVYGGETK	-0.95 -1.01 -0.92	[-0.72, -1.33] [-0.8, -1.4] [-0.8, -1.41]	** ** **	100.0 95.0 95.0
*P01871*	Ig mu chain C region	IGHM	GFPSVLR ESGPTTYK	-1.34 -1.12	[-1.14, -2.03] [-1.15, -2.11]	** **	95.0 94.7
*Q86VB7*	Scavenger receptor cysteine-rich type 1 protein M130	C163A	LVDGVTECSGR INPASLDK	-0.34 -0.34	[-0.25, -0.52] [-0.21, -0.57]	** **	95.0 90.0
*P00738*	Haptoglobin	HPT	DYAEVGR	-0.95	[-0.7, -1.56]	**	95.0
*Q9UHL4*	Dipeptidyl peptidase 2	DPP2	DLFLQGAYDTVR SLPFGAQSTQR DVTADFEGQSPK	0.26 0.22 0.22	[0.2, 0.46] [0.18, 0.47] [0.15, 0.43]	** ** **	95.0 89.5 89.5
*Q08431*	Lactadherin	MFGE8	VTFLGLQHWVPELAR NLFETPILAR	0.43 0.40	[0.27, 0.54] [0.26, 0.52]	** **	92.3 90.0
*O14791*	Apolipoprotein L1	APOL1	VAQELEEK ALADGVQK	-0.58 -0.64	[-0.38, -0.76] [-0.38, -0.78]	** **	89.5 89.5
*P35858*	Insulin-like growth factor-binding protein complex acid labile subunit	ALS	VAGLLEDTFPGLLGLR LAYLQPALFSGLAELR	-0.47 -0.31	[-0.22, -0.56] [-0.15, -0.53]	** **	80.0 76.5
*Q9Y6R7*	IgGFc-binding protein	FCGBP	GATTSPGVYELSSR FYPAGDVLR	-0.29 -0.24	[-0.15, -0.46] [-0.15, -0.5]	** **	80.0 75.0
*P01011*	Alpha-1-antichymotrypsin	AACT	AVLDVFEEGTEASAATAVK	-0.23	[-0.14, -0.4]	**	80.0
*P01619*	Ig kappa chain V-III region B6	KV320	LLIYGASSR	-0.61	[-0.27, -0.81]	**	80.0
*P00338*	L-lactate dehydrogenase A chain	LDHA	FIIPNVVK	0.25	[0.14, 0.42]	**	80.0
*Q86UN3*	Reticulon-4 receptor-like 2	R4RL2	HLQALEELDLGDNR LFLQNNLIR	0.23 0.21	[0.11, 0.37] [0.08, 0.33]	** **	75.0 70.0
*P40925*	Malate dehydrogenase, cytoplasmic	MDHC	VIVVGNPANTNCLTASK NVIIWGNHSSTQYPDVNHAK	0.27 0.28	[0.13, 0.48] [0.14, 0.5]	** **	75.0 70.0
*Q6MZW2*	Follistatin-related protein 4	FSTL4	DSGLFGQYLLTPAR VLQSIGVDPLPAK LLVESLFR	0.20 0.18 0.20	[0.07, 0.35] [0.06, 0.34] [0.03, 0.31]	* * *	75.0 75.0 75.0
*P07195*	L-lactate dehydrogenase B chain	LDHB	GLTSVINQK FIIPQIVK IVVVTAGVR	0.11 0.18 0.14	[0.04, 0.25] [0.05, 0.33] [0.07, 0.29]	* * **	75.0 75.0 70.0
*Q9NS98*	Semaphorin-3G	SEM3G	LFLGGLDALYSLR DYPDEVLQFAR	0.10 0.05	[0.03, 0.33] [0.05, 0.32]	* *	75.0 73.7
*Q15782*	Chitinase-3-like protein 2	CHI3L2	LVCYFTNWSQDR	-0.35	[-0.04, -0.78]	ns	75.0
*Q9NT99*	Leucine-rich repeat-containing protein 4B	LRC4B	DLAEVPASIPVNTR	0.16	[0.08, 0.28]	**	70.0
*P48058*	Glutamate receptor 4	GRIA4	NTDQEYTAFR	0.28	[0.07, 0.39]	*	69.2

Ordered by the percentage of patients where the peptide was changed.

^a^ % of patients with protein increased/decreased (log_2_ FC >0/<0) in direction of median.

* FDR <0.05

** FDR<0.01, ns = not significant.

**Table 6:** Verification of proteins changed by natalizumab treatment, of the most relevant CSF-PR categories.

*Category*	CSF- PR *RRMS*	Discovery *Treatment*	*Measured Proteins* *PRM-MS*	*Verified total*	*Verified* *proteins (Gene names)*	*Partially verified* *	*Not* *verified*
					VCAM1,		
	**↑**	↓	6	5	FCGBP, C2,	-	CHI3L2
					CHI3L1, B2M		
*Opposite*	**↓**	↑	15	9	APP, MFGE8, R4RL2, FSTL4, CNTP2, PGCB, NRX2A, L1CAM, GRIA4	NRX1A, NRX3A	KLK6, NRCAM, SLITRK1, NRX1A
*Equal*	**↑**	↑	0	0	-	-	-
	**↓**	↓	3	3	GC, APOA1, CFB	-	-

* When several surrogate peptides were measured, at least one was significantly different (FDR<0.05) in the direction of the discovery experiment.

The proteins that decreased the most after treatment in the verification experiment were IGJ, VCAM1, HPT, IGM, C163 and APOL1, each having peptides that decreased in 90% of the patients. Of these, IGHM had the greatest decrease (log_2_ median FC of -1.34 and -1.12 for the measured peptides, respectively). This is similar to the decrease of the protein measured in the proteomic discovery study (log_2_ median FC -1.4). MFGE8 and DPP2 were confirmed to increase in abundance in over 80% of the verification samples.

The remaining candidates increasing in the discovery study also increased significantly, but in a lower percentage of the samples. This may imply that the decrease in immune-related proteins were more common following treatment than the increase in proteins associated with metabolic and neurological activity. It may also reflect the modest abundance change seen in the latter categories.

### Differences in protein concentration between baseline and follow-up sample

Note that the protein concentration was significantly different (two-sided paired *t*-test *p*-value << 0.01) between the samples collected at baseline and the follow-up sample included in the discovery study (but not in the verification study). Principal component analysis (PCA) of the raw values in the discovery study through the MaxQuant quality control program PTXQC [32] showed no clear grouping of the baseline and follow-up sample, and comparable number of identifications were observed across runs in the final dataset as observable in Perseus [33]. Thus, there does not seem to be any global biases between baseline and follow-up samples.

## Discussion

### Proteins highly decreased in abundance after treatment

Our analyses confirmed the well documented anti-inflammatory effect of natalizumab [17, 34–36], with a decrease in immune response proteins like the complement, immunoglobulins (IgM, IgA and IgG) and CD163 after treatment. These proteins have previously been reported as increased (and some not affected) in RRMS. Immunoglobulin and complement activation are commonly seen in active demyelinating lesions [17, 37]. Thus, the most affected pathways for those significantly differentially abundant proteins that have been reported increased in RRMS patients compared to controls, and that were decreased after treatment of natalizumab in our study, belonged to the two categories immune response and protein activation cascade.

IgG oligoclonal bands (OCBs) are observed in most RRMS patients [38]. However, OCBs from IgM are rarer, and intrathecal IgM synthesis has in some studies shown a prognostic value in conversion from clinically isolated syndrome (CIS) to clinically definite MS (CDMS), as well as marker for a more severe disease course [39]. As natalizumab is administered to treat highly active RRMS, this can explain the reduction observed in IgM. That the majority of the immunoglobulins were significantly reduced by the treatment is in line with previous reports of disappearance of CSF-OCB in patients receiving natalizumab [34, 40]. The Ig kappa light chains have been proposed as prognostic biomarkers in MS and were decreased after treatment [41, 42].

The chitinase family, predominantly chitinase 3-like protein 1 (CHI3L1), has received widespread attention as possible biomarkers in a multitude of neurological disorders, including RRMS, as previously reviewed [43]. This protein has been shown to increase with disease severity in RRMS and PPMS, and is a possible prognostic marker for the conversion from CIS to CDMS [43, 44]. We observed a decrease in CHI3L1 during natalizumab therapy, supporting previous proteomics studies [17]. CHIT1 is expressed in macrophages and monocytes [43], and CHIT1 levels have been shown to predict disease severity and discriminate between chronic active and chronic inactive lesions in a recent study [45].

### Proteins highly increased in abundance after treatment

Our results also showed that proteins associated with metabolism and CNS function increased after treatment. This included an increase in both lactate dehydrogenase A and B (LDHA and LDHB), as well as malate dehydrogenase (MDH1), which to our knowledge has not previously been reported to be affected by treatment. The LDH functional protein has been found to have increased activity in plasma from SPMS patients compared to healthy controls [46], and abnormal isoenzyme types of LDH have been found in the CSF of MS patients [47]. Further investigation of the LDH isoenzyme could reveal what effect natalizumab has on lactate metabolism during treatment.

Lactate in the brain interstitial fluid has been used as a marker for neuronal activity [48], and CSF-lactate have been found to be a potential prognostic marker for disease progression in RRMS as it correlated with neurofilament light protein [49]. Furthermore, serum lactate levels correlated with radiological findings and disability scores, suggesting mitochondrial dysfunction in MS [50]. Thus, measuring lactate in patients receiving natalizumab treatment could be of interest as a possible monitoring biomarker.

### Functional analysis shows changes in disease-relevant categories

APP, LRRC4B, and neurexin 1–3, among others, increased during natalizumab therapy. These are among the proteins that previously have been reported decreased in RRMS patients compared to controls. This category was enriched in GOBP terms *nervous system development* and *generation of neurons*, including plasma membrane bounded cell projection morphogenesis, neuron projection morphogenesis, neurogenesis, neuron differentiation, neuron cell-cell adhesion, neuron projection development and axon development. Thus, our findings may indicate beneficial repair processes induced by the natalizumab treatment.

The beta-amyloid peptide of APP has been suggested as a marker for neurodegeneration in MS as it was used to predict disease progression as measured by disability score [51, 52]. Furthermore, it has been shown to be a marker of myelin damage as normal-appearing white matter correlated positively with absolute concentrations in the CSF [53], and APP is elevated in axons surrounding MS plaques [54]. In our study, APP was increased by treatment in the majority of patients (79% and 70% in the discovery and verification experiments, respectively), supporting a role for this protein in MS. As an increase in beta-amyloid is inversely correlated to disease progression, the increase in APP in the CSF could similarly be beneficial, however this needs confirmation.

It has previously been reported that some of the proteins linked to neurodegeneration differed in abundance in patients six months after treatment with natalizumab, but not twelve months after treatment [55]. These proteins were alpha-1 antichymotrypsin (AACT), contactin-1 (CNTN1), neuronal cell adhesion molecule (NRCAM), and neural cell adhesion molecule 1. Both AACT and NRCAM were changed in our discovery study, but only the change for AACT was confirmed in our verification study. Stoop *et al*. reported that they did not detect any proteins previously related to neurodegeneration confidently changed in their study of patients after 12 months of natalizumab treatment [17]. Furthermore, they also did not detect any increased proteins after the treatment. One reason that we found a relatively large number of proteins increased after natalizumab treatment compared to Stoop *et al*. could be the highly increased depth of proteome coverage (1164 vs. 578 unique proteins), revealing more of the less abundant proteins involved in neurodegeneration. Another reason could be the higher number of patients included in our study (n = 59 vs. n = 17) leading to increased power allowing us to find smaller differences. Thus, to our knowledge, the changes in proteins with neurological activity observed in this study provide novel information on the effect of natalizumab treatment in RRMS.

In total, 19 proteins previously reported as reduced/increased in RRMS were also reduced/increased during treatment in our data. Seven of these were all part of the same STRING network with the main biological processes of *steroid metabolic process*. In this category we find complement factor B, vitamin D-binding protein, alpha B-1-glycoprotein, and the lipoproteins APOD, APOA1 and APOA2. Lower serum ApoA-II and ApoA-I levels have been associated with greater neuroaxonal injury as measured by CSF-NfL [56]. These proteins are likely to be affected by MS and continue to be affected in the same way even after natalizumab treatment. This may indicate that neuroaxonal injury is still ongoing in these patients even after natalizumab therapy. This could mean that even if inflammation apparently is heavily reduced, other disease mechanisms are still ongoing–such as those associated with chronic active lesions, independent of clinical relapse activity and new MRI lesions activity [57, 58].

### Verification of proteins changed by natalizumab treatment

In the verification study, the decrease of the proteins IGHM, VCAM1 and IGJ was observed in more than 90% of the patients. Of the most changed proteins in the discovery study, only CHI3L2 was not verified to change due to the treatment. That a high number of proteins was verified in new samples, shows that the data from the discovery study to a large degree can be reproduced in another patient cohort with a different methodological approach. The few proteins that were not verified, were changed in the same direction as in the discovery study, but not significantly. Furthermore, verification of unchanged proteins from the discovery study underlines the reproducibility in sample preparation and data analysis.

Proteins of relevance to processes changed in comparison to online datasets were included in the PRM verification. This further confirmed the decrease in inflammatory associated proteins, and the increase of metabolic and CNS-associated proteins. In contrast to the decrease in inflammatory-associated proteins, the increase of proteins associated with metabolic and CNS activity was not seen in as high a percentage of patients, indicating that this is an effect of the treatment that is more variable than the reduction of inflammatory activity.

### Treatment changed the abundance of known biomarker candidates

As the verification of biomarker candidates is an ongoing and highly interesting topic in MS, a summary of how these proteins were affected by natalizumab therapy deserves a special focus. Of the proposed biomarkers in MS [59], IgM, CHI3L1 and CD163 were decreased by the treatment (and verified by PRM). CHIT1 was most changed by treatment in the discovery study but could not be measured by PRM. CHI3L2 was decreased in the discovery study, but could not be verified by PRM, whereas the surface markers CD14 and TREM2 were not among the most changed proteins in our findings.

From the proposed treatment response markers for natalizumab treatment [60], our study confirms that CHI3L1 is significantly reduced by the treatment in the majority of patients (approximately 70% in both discovery and verification). Of the remaining markers, osteopontin (OSTP) was detected as decreased in the discovery study but not significantly different. Finally, the suggested biomarkers fetuin-A, CXCL13 and NFL/NFH were not detected in the dataset.

## Conclusion

This study confirms that a large number of biomarker candidates relevant for MS and treatment response are affected by natalizumab treatment. It also suggests new proteins and processes that ought to be explored further to possibly disclose novel disease mechanisms or predict treatment response. Investigating whether some of the protein changes observed in this study correspond to clinical or magnetic resonance imaging (MRI) parameters during disease progression could provide valuable information related to disease mechanisms and potential novel biomarkers, but this was not a focus in this study.

Furthermore, the observed changes were often quite small but nevertheless significant. Since paired samples from the same individual were investigated, inter-individual differences in the CSF proteome were removed, allowing for the detection of smaller changes. Thus, repeated follow-up sampling of CSF could provide even more accurate information concerning treatment effects. However, given the challenges related to CSF sampling, follow-up studies in more easily available samples, preferably blood, could be more realistic to use for evaluating clinical application.

Finally, it is important to note that many of the proteins found as changed in our study are not unique for the comparison of the treatment effect of natalizumab nor for RRMS compared to controls but are also suggested in studies of other CNS diseases. This indicates that a disbalance in some of these proteins could perhaps either be a cause or an effect of CNS disease in general. Currently, different CNS diseases are often investigated individually, however, it would be highly relevant to investigate potential biomarkers across a multitude of neurological diseases to better understand their role in the CNS pathology.

## Supporting information

S1 Fig The distribution of the median fold-changes during treatment were greatly affected by immunoglobulins that are target for somatic rearrangement (TSR-immunoglobulins) (antibodies). (PNG)

S1 Table Comparison of proteins affected/not affected by the treatment to public data comparing RRMS to neurological controls in CSF-PR. (PNG)

S2 Table Verification of proteins changed by natalizumab treatment, sorted into the CSF- PR categories. (PNG)

S3 Table Peptides and proteins measured by PRM-MS, ordered by gene name. (PDF)
