# Supplementary figures and images for: Natalizumab promotes anti-inflammatory and repair effects in multiple sclerosis
Source: PLoS One. 2024 Mar 25;19(3):e0300914. doi: 10.1371/journal.pone.0300914 (PMC10962820; doi:10.1371/journal.pone.0300914)

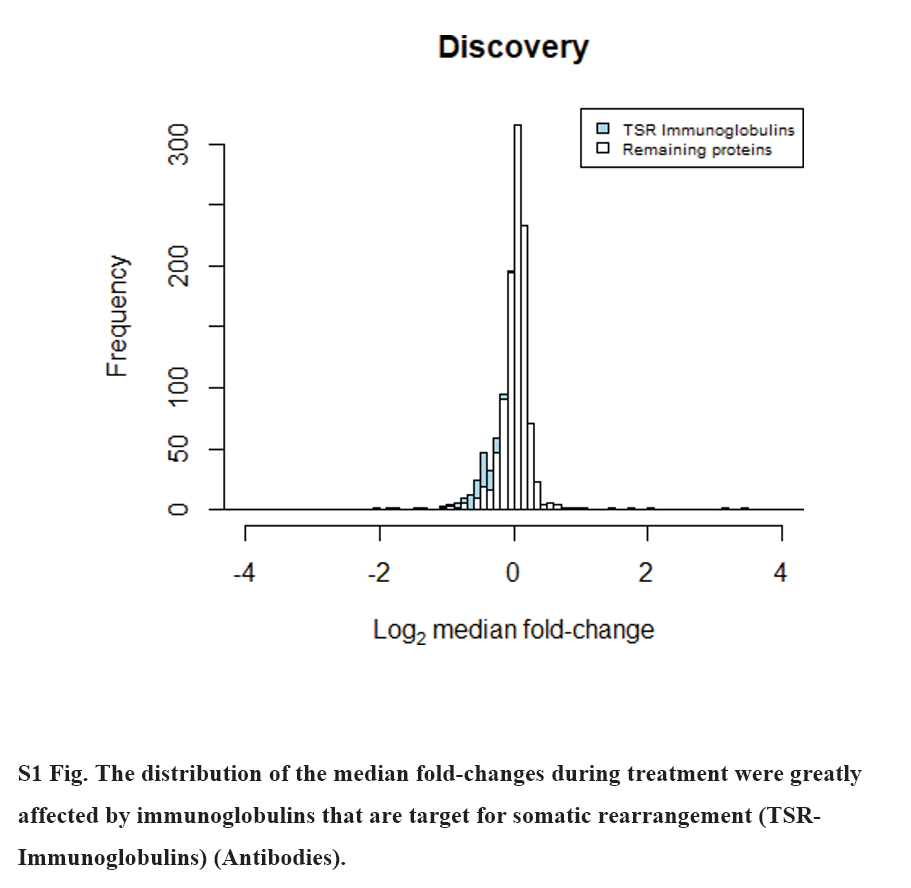

Supplement: S1 Fig — (PNG) [file pone.0300914.s001.png]

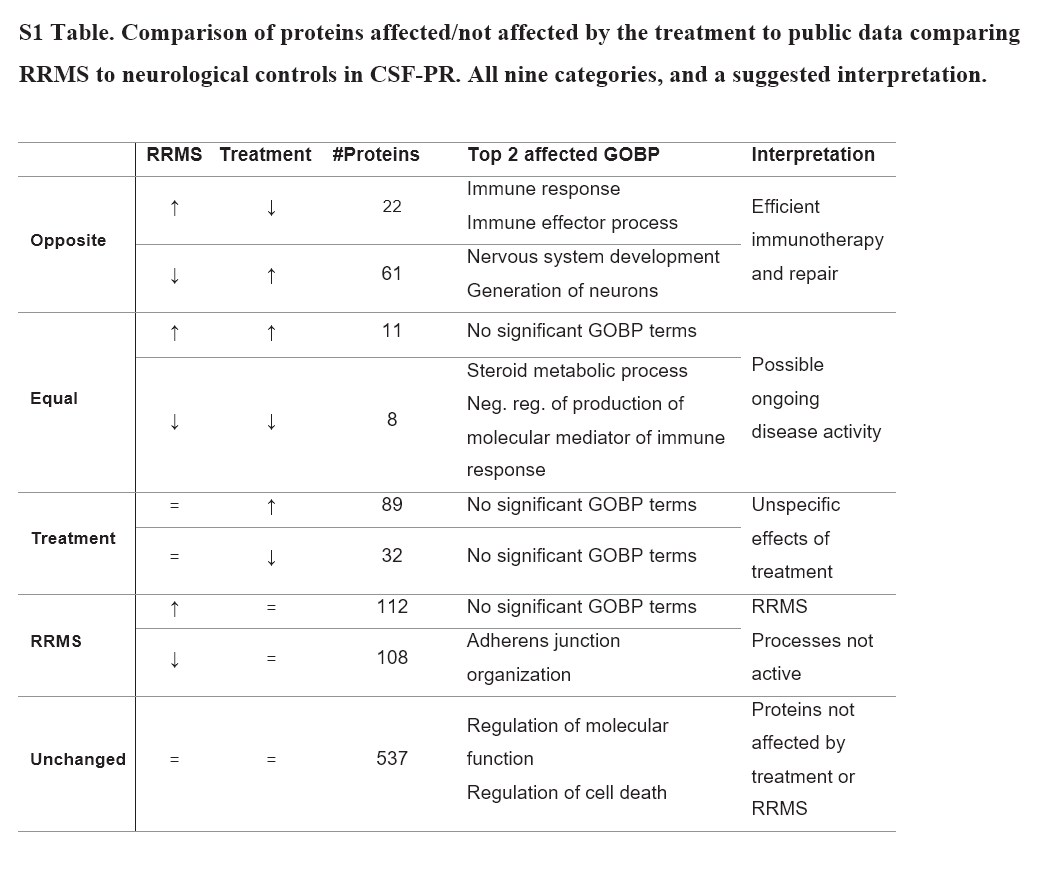

Supplement: S1 Table — (PNG) [file pone.0300914.s002.png]

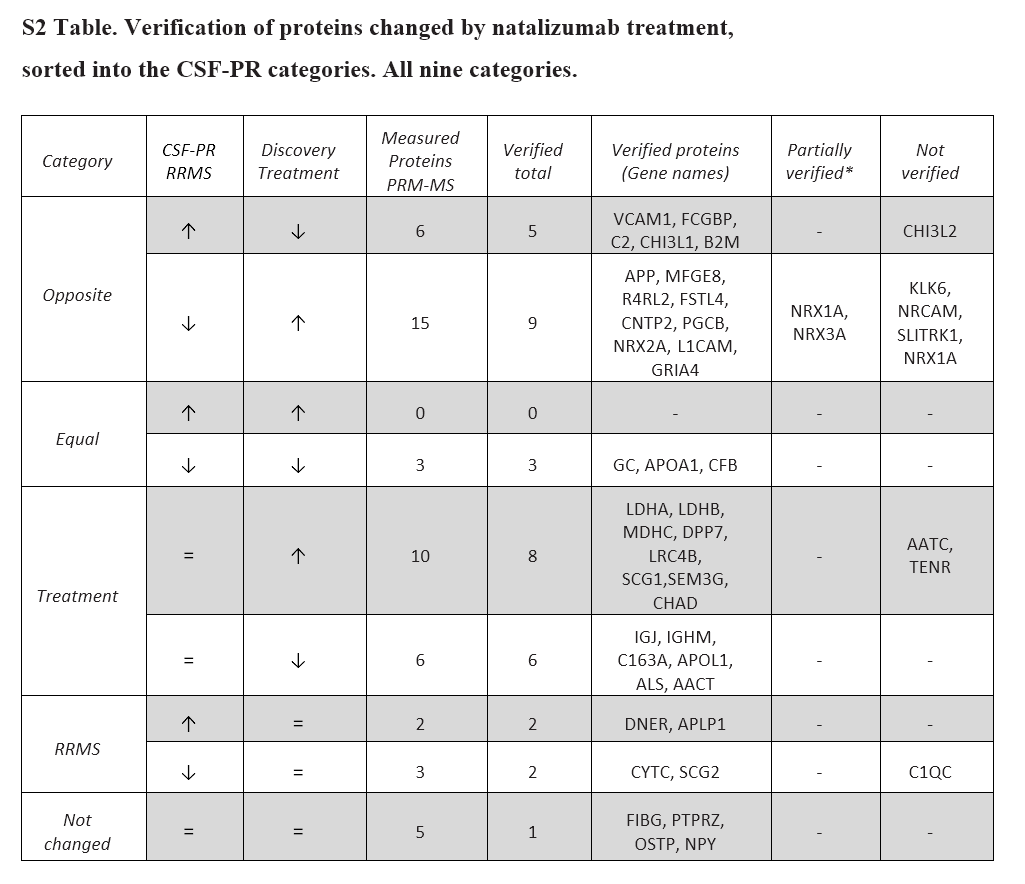

Supplement: S2 Table — (PNG) [file pone.0300914.s003.png]
