# Supplementary material for: Natalizumab promotes anti-inflammatory and repair effects in multiple sclerosis
Source: PLoS One. 2024 Mar 25;19(3):e0300914. doi: 10.1371/journal.pone.0300914 (PMC10962820; doi:10.1371/journal.pone.0300914)
Supplement: S3 Table — (PDF) [file pone.0300914.s004.pdf]

S3 Table. Peptides and proteins measured by PRM-MS, ordered by gene name. The FDR status without removal of the outlier patient is included in the last column, and differences in the verification status between the two datasets are highlighted (last column) (blank= same verification status in both datasets).

Accession	Gene	CSF-PR	Peptides	p-value	FDR <0.05	% Blank	Median log2 FC	% Changed	95% CI	FDR<0.05, all samples
P01011	AACT	Natalizumab only	AVLDVFEEGTEASAATAVK	0.00	TRUE	0	-0.23	80	[-0.14, -0.4]	
P17174	AATC	Natalizumab only	LALGDDSPALK	0.05	FALSE	0	0.14	60	[0, 0.23]	
			ITWSNPPAQGAR	0.09	FALSE	0	0.05	60	[-0.01, 0.2]	
P35858	ALS	Natalizumab only	VAGLLEDTFPGLLGLR	0.00	TRUE	0	-0.47	80	[-0.56, -0.22]	
			LAYLQPALFSGLAELR	0.00	TRUE	15	-0.31	76	[-0.53, -0.15]	
P51693	APLP1	RRMS only	WEPDPQR	0.57	FALSE	0	0.11	55	[-0.08, 0.14]	
			DELAPAGTGVSR	0.75	FALSE	0	0.01	55	[-0.07, 0.1]	
P02647	APOA1	Equal	THLAPYSDELR	0.00	TRUE	0	-0.35	80	[-0.46, -0.17]	
O14791	APOL1	Natalizumab only	VAQELEEK	0.00	TRUE	5	-0.58	89	[-0.76,-0.38]	
			ALADGVQK	0.00	TRUE	5	-0.64	89	[-0.78,-0.38]	
P05067	APP	Opposite	VESLEQEAANER	0.02	TRUE	0	0.16	70	[0.05,0.4]	
P61769	B2M	Opposite	VNHVTLSQPK	0.00	TRUE	0	-0.29	70	[-0.4,-0.11]	FALSE
Q86VB7	C163A	Natalizumab only	LVDGVTECSGR	0.00	TRUE	0	-0.34	95	[-0.52,-0.25]	
			INPASLDK	0.00	TRUE	0	-0.34	90	[-0.57,-0.21]	
P02747	C1QC	RRMS only	FQSVFTVTR	0.01	TRUE	0	-0.25	85	[-0.42,-0.08]	FALSE
			TNQVNSGGVLLR	0.02	TRUE	0	-0.18	75	[-0.43,-0.05]	FALSE
P00751	CFAB	Equal	EELLPAQDIK	0.01	TRUE	0	-0.30	80	[-0.54,-0.1]	FALSE
P36222	CHI3L1	Opposite	TLLSVGGWNFGSQR	0.01	TRUE	0	-0.27	70	[-0.53,-0.07]	FALSE
Q15782	CHI3L2	Opposite	LVCYFTNWSQDR	0.10	FALSE	40	-0.35	75	[-0.78,-0.04]	
O15335	CHAD	Natalizumab only	FSDGAFLGVTTLK	0.01	TRUE	25	0.20	73	[0.09,0.38]	
			NQLSSYPSAALSK	0.02	TRUE	25	0.14	73	[0.08,0.41]	
Q9UHC6	CNTP2	Opposite	VQFNQIAPLK	0.01	TRUE	0	0.19	70	[0.06,0.3]	
P06681	CO2	Opposite	HAIILLTDGK	0.00	TRUE	0	-0.26	80	[-0.46,-0.11]	FALSE
P01034	CYTC	RRMS only	ALDFAVGEYNK	0.68	FALSE	0	-0.01	50	[-0.12,0.08]	
Q8NFT8	DNER	RRMS only	VTATGFQQCSLIDGR	0.15	FALSE	10	0.10	56	[-0.02,0.15]	
Q9UHL4	DPP2	Natalizumab only	DLFLQGAYDTVR	0.00	TRUE	0	0.26	95	[0.2,0.46]	
			SLPFGAQSTQR	0.00	TRUE	5	0.22	89	[0.18,0.47]	
			DVTADFEGQSPK	0.00	TRUE	5	0.22	89	[0.15,0.43]	
Q9Y6R7	FCGBP	Opposite	FYPAGDVLR	0.00	TRUE	0	-0.24	75	[-0.5,-0.15]	
			GATTSPGVYELSSR	0.00	TRUE	0	-0.29	80	[-0.46,-0.15]	
P02679	FIBG	Not Changed any	YEASILTHDSSIR	0.15	FALSE	5	-0.27	58	[-0.44,0.07]	
Q6MZW2	FSTL4	Opposite	DSGLFGQYLLTPAR	0.01	TRUE	0	0.20	75	[0.07,0.35]	
			LLVESLFR	0.02	TRUE	0	0.20	75	[0.03,0.31]	
			VLQSIGVDPLPAK	0.01	TRUE	0	0.18	75	[0.06,0.34]	

P02774	GC	Equal	HLSLLTTLSNR	0.00	TRUE	0	-0.35	80	[-0.47,-0.14]	FALSE
P48058	GRIA4	Opposite	NTDQEYTAFR	0.03	TRUE	35	0.28	69	[0.07,0.39]	
P00738	HP	Natalizumab only	DYAEVGR	0.00	TRUE	0	-0.95	95	[-1.56,-0.7]	
P0DOX5	IGG1		DTLMISR	0.00	TRUE	0	-0.47	85	[-0.74,-0.3]	
P01871	IGHM	Natalizumab only	GFPSVLR	0.00	TRUE	0	-1.34	95	[-2.03,-1.14]	
			ESGPTTYK	0.00	TRUE	5	-1.12	95	[-2.11,-1.15]	
P01824, P0DP08	IGHV4-39, IGHV4-38-2		VTISVDTSK	0.00	TRUE	0	-0.40	95	[-0.8,-0.31]	
P01591	IGJ	Natalizumab only	SSEDPNEDIVER	0.00	TRUE	15	-0.95	100	[-1.33,-0.72]	
			IVLVDNK	0.00	TRUE	0	-1.01	95	[-1.4,-0.8]	
			CYTAVVPLVYGGETK	0.00	TRUE	0	-0.92	95	[-1.41,-0.8]	
P0DOX7	IGK		ASSLESGVPSR	0.00	TRUE	0	-0.49	95	[-0.98,-0.34]	
Q92876	KLK6	Opposite	LSELIQPLPLER	0.06	FALSE	0	0.04	55	[0,0.22]	
P01619	KV320		LLIYGASSR	0.00	TRUE	0	-0.61	80	[-0.81,-0.27]	
P32004	L1CAM	Opposite	AQLLVVGSPGPVPR	0.03	TRUE	5	0.10	68	[0.02,0.2]	FALSE
P00338	LDHA	Natalizumab only	FIIPNVVK	0.00	TRUE	0	0.25	80	[0.14,0.42]	
P07195	LDHB	Natalizumab only	GLTSVINQK	0.01	TRUE	0	0.11	75	[0.04,0.25]	
			FIIPQIVK	0.01	TRUE	0	0.18	75	[0.05,0.33]	
			IVVVTAGVR	0.00	TRUE	0	0.14	70	[0.07,0.29]	
Q9NT99	LRC4B	Natalizumab only	DLAEVPASIPVNTR	0.00	TRUE	0	0.16	70	[0.08,0.28]	
P40925	MDHC	Natalizumab only	VIVVGNPANTNCLTASK	0.00	TRUE	0	0.27	75	[0.13,0.48]	
			NVIIWGNHSSTQYPDVNHAK	0.00	TRUE	0	0.28	70	[0.14,0.5]	
Q08431	MFGE8	Opposite	VTFLGLQHWVPELAR	0.00	TRUE	35	0.43	92	[0.27,0.54]	
			NLFETPILAR	0.00	TRUE	0	0.40	90	[0.26,0.52]	
P01303	NPY	Not Changed any	ESTENVPR	0.66	FALSE	0	0.05	60	[-0.15,0.23]	
Q92823	NRCAM	Opposite	SLPSEASEQYLTK	0.31	FALSE	0	0.05	55	[-0.04,0.13]	
			VFNTPEGVPSAPSSLK	0.19	FALSE	0	0.05	65	[-0.04,0.2]	
Q9ULB1	NRX1A	Opposite	DLFIDGQSK	0.03	TRUE	0	0.10	70	[0.01,0.21]	FALSE
			SDLYIGGVAK	0.14	FALSE	0	0.07	60	[-0.03,0.18]	
Q9P2S2	NRX2A	Opposite	LSALTLSTVK	0.01	TRUE	0	0.12	75	[0.04,0.24]	
Q9Y4C0	NRX3A	Opposite	SDLSFQFK	0.01	TRUE	0	0.22	65	[0.06,0.33]	
			ANDGEWYHVDIQR	0.09	FALSE	10	0.16	67	[-0.01,0.22]	
P10451	OSTP	Not Changed any	AIPVAQDLNAPSDWDSR	0.62	FALSE	25	0.08	53	[-0.14,0.24]	
Q96GW7	PGCB	Opposite	FNVYCFR	0.01	TRUE	35	0.14	69	[0.09,0.33]	
P00747	PLMN	Not Changed any	LSSPAVITDK	0.00	TRUE	0	-0.27	75	[-0.41,-0.1]	
P23471	PTPRZ	Not Changed any	AIIDGVESVSR	0.26	FALSE	0	0.05	55	[-0.04,0.15]	
Q86UN3	R4RL2	Opposite	HLQALEELDLGDNR	0.00	TRUE	0	0.23	75	[0.11,0.37]	
			LFLQNNLIR	0.00	TRUE	0	0.21	70	[0.08,0.33]	
P05060	SCG1	Natalizumab only	GEAGAPGEEDIQGPTK	0.01	TRUE	0	0.08	75	[0.04,0.26]	
P13521	SCG2	RRMS only	ALEYIENLR	0.81	FALSE	0	-0.02	60	[-0.14,0.11]	

Q9NS98	SEM3G	Natalizumab only	LFLGGLDALYSLR	0.02	TRUE	0	0.10	75	[0.03,0.33]	
			DYPDEVLQFAR	0.01	TRUE	5	0.05	74	[0.05,0.32]	
Q96PX8	SLITRK1	Opposite	LSNVQELFLR	0.14	FALSE	0	0.08	55	[-0.03,0.18]	
Q92752	TENR	Natalizumab only	YEVSVSAVR	0.08	FALSE	5	0.11	63	[-0.01,0.21]	
P19320	VCAM1	Opposite	SLEVTFTPVIEDIGK	0.00	TRUE	0	-0.40	95	[-0.58,-0.28]	
			LTAFPSESVK	0.00	TRUE	0	-0.44	100	[-0.65,-0.35]	
